# hPG_80_ (circulating progastrin) as a blood biomarker for high-grade glial tumors: A pilot study

**DOI:** 10.3389/fneur.2022.1073476

**Published:** 2023-01-11

**Authors:** Melanie Casile, Judith Passildas, Bérengère Vire, Ioana Molnar, Xavier Durando

**Affiliations:** ^1^INSERM U1240 IMoST, University of Clermont Auvergne, Clermont-Ferrand, France; ^2^UMR 501, Clinical Investigation Centre, Clermont-Ferrand, France; ^3^Clinical Research and Innovation Department, Centre Jean Perrin, Clermont-Ferrand, France; ^4^BIODENA CARE, Montpellier, France; ^5^Oncology Department, Centre Jean Perrin, Clermont-Ferrand, France

**Keywords:** progastrin, hPG_80_, high grade glial tumors, biomarker, brain cancer

## Abstract

**Background:**

Currently, the long-term prognosis and survival rate of patients with high-grade glial tumors remains poor and there are no biomarkers. hPG_80_ (circulating progastrin) secreted into the blood by tumor cells has been widely studied in colorectal cancer. Its involvement in tumorigenesis has been demonstrated in the literature. Moreover, according to a recent study, hPG_80_ is expressed in the blood of cancer patients at a significantly higher concentration than in the control group composed of healthy blood donors.

**Methods:**

The PROGLIO study is a pilot, single-center, longitudinal study that primarily seeks to evaluate circulating plasma hPG_80_ concentrations over time in patients with high-grade glial tumors. A fasting blood sample will be taken on the start and end day of radiotherapy and during the adjuvant chemotherapy (every 3 cycles). Follow-up monitoring will be performed for 9 months, with a blood sample taken every 3 months on the day of the follow-up MRI. The study plans to recruit 30 patients and recruitment started in February 2022.

**Trial registration:**

ClinicalTrials.gov, ID NCT05157594; registered on October 27, 2021.

## 1. Introduction

Since 2016, glial tumors have been classified *via* a new approach, which combines histo-pathological and molecular features (1). This classification has been recently updated. The 2021 fifth edition introduces major changes that advance the role of molecular diagnostics in central nervous system tumor classification (2). In addition, this classification enables the aggressiveness of the tumor to be assessed. The most aggressive tumors are classified as high-grade gliomas, i.e., WHO grade 3 and 4, and have a poor prognosis with an estimated 5-year survival of 30% for grade 3 and < 5% for grade 4 (3). Indeed, the long-term prognosis and survival rate for patients with high-grade glial tumors remains poor (4).

The *GAST* gene codes for a 101-amino acid precursor peptide, preprogastrin, which generates progastrin after several post-translational modifications. This 80-amino acid pro-hormone is then cleaved by pro-hormone convertases and carboxypeptidases to form glycine extended gastrins (G34-Gly and G17-Gly). These glycine ends then serve as substrates for peptidyl-α-amidating monooxygenases, resulting in the formation of the two biologically active majority forms of gastrin in the G-cells of the antrum of the stomach, controlling acid secretions during digestion: G34-NH2 for 34-amino acid gastrin and G17-NH2 for 17-amino acid gastrin (5, 6). In a pathological situation, the disruption of the Wnt/ß-catenin pathway in cancer induces the activation of the expression of the *GAST* gene coding for progastrin and its secretion in circulating form known as hPG_80_ (7). Its role has been widely studied in colorectal cancer. Thus, its involvement in the formation of cell junctions, in the inhibition of apoptosis, in the regulation of cancer stem cells, in angiogenesis and in the regulation of the Wnt signaling pathway, has been demonstrated in the literature (8–13). Furthermore, progastrin has been shown to be a factor in radio-resistance (14). An increase in *GAST* gene expression has been observed after irradiation. On the other hand, targeting hPG_80_ induces an increase in radiation-induced apoptosis. A decrease in hPG_80_ expression increases radiation-induced DNA damage and leads to the inhibition of the AKT and ERK pathways, induced by irradiation (14).

A prospective study, which was analyzed retrospectively, was performed on a total of 1,546 patients and 557 healthy blood donors, enabling the determination of hPG_80_, with the DxPG80 IVD ELISA kit developed by BIODENA CARE (15). In this study, the levels of hPG_80_ (circulating progastrin) were determined in 11 different epithelial and non-epithelial cancers (colorectal, breast, uterus, ovary, prostate, kidney, pancreas, esophagus/stomach, liver, melanoma and lung). Median hPG_80_ concentrations across cancer types were all above the limit for detection (1.2 pM) and the limit for quantification (2.3 pM). When all the cancer types were combined, the median hPG_80_ concentration was 4.88 pM. The median hPG_80_ concentration in the healthy blood donors was 1.05 pM. This study demonstrates that hPG_80_ is expressed in the blood of patients with all types of cancers at significantly higher concentrations than in a control group of healthy blood donors (7). In addition, a retrospective study of 143 patients with metastatic kidney cancer found that hPG_80_ was detected in 94% of the patients (16). More recently, a retrospective study of 168 patients with hepatocellular carcinoma found that hPG_80_ was detected in 84% of the patients (17).

As hPG_80_ is involved in many signaling pathways, which are themselves involved in the tumorigenesis of many different types of cancers, it is justifiable to assume that hPG_80_ will be measurable in patients with high-grade brain tumors. In addition, BIODENA CARE has preliminary data on IDW-wild type glioblastoma treated with radio-chemotherapy, published on a poster at the annual ASCO meeting this year (18). After surgery, hPG80 was detected in 48 (69%) patients (hPG80+) with a median concentration of 9.52 pM (IQR 5.21–21.20). This data confirms the detection of hPG_80_ in these tumors and a decrease in that marker after total tumor resection. This data provides a strong basis to reasonably hypothesize that hPG_80_ is measurable in the blood of patients with high-grade brain tumors. Furthermore, there is no routine use of a blood biomarker for glial tumors. This justifies the interest of conducting a study assessing plasma hPG_80_ concentrations in patients with high-grade brain tumors.

## 2. Methods and analysis

### 2.1. Study design

The PROGLIO study is a pilot, single-center, longitudinal study aiming to evaluate plasma hPG_80_ concentrations over time in patients with high-grade brain tumors.

This study has been registered on Clinicaltrials.gov (NCT05157594). A maximum of thirty patients are expected to be enroled. The study was started in February 2022 with an 18-month enrolment period and an estimated completion date by February 2025.

### 2.2. Study objectives and endpoints

#### 2.2.1. Main objective and endpoint

The main objective of the study is to evaluate the plasma levels of hPG_80_ (circulating progastrin) during therapeutic follow-up and relapse monitoring among patients with high-grade glial tumors.

The primary endpoint is hPG_80_ plasma concentrations measured by BIODENA CARE using their DxPG80 IVD ELISA kit, from inclusion to the last follow-up.

#### 2.2.2. Secondary objectives

The secondary objectives are:

– To study hPG_80_ concentrations according to tumor progression using the Response Assessment in Neuro-Oncology (RANO) criteria, which take progression objectified by imagery, clinical status and level of steroid therapy into account.
– To evaluate the sensitivity to irradiation of hPG_80_ concentration variations.
– To compare hPG_80_ plasma levels in patients, and to study inter-individual variations before, after and during disease management.

### 2.3. Participant eligibility

The inclusion and non-inclusion criteria are presented in Table 1.

**Table 1:** Section criteria.

**Inclusion criteria**	**Non-inclusion criteria**
Female or male 18 years of age or older.	Patient under guardianship or curatorship.
Patients with a high-grade primary glial tumor (Astrocytoma grade 3; Glioblastoma; Anaplastic Oligodendroglioma).	Psychological disorder *(cognitive disorders, vigilance disorders, etc.)* or social reasons *(deprivation of liberty by judicial or administrative decision)* or geographical reasons that could compromise the medical follow-up of the trial.
Patients scheduled to begin radiotherapy and/or chemotherapy.	Refusal to participate.
Able to give informed consent to participate in the research.	Pregnant or breastfeeding woman.

Patients with a high-grade glial tumor (glioblastomas IDH-wt, astrocytomas IDH mutated grade 3 and 4, oligodendrogliomas grade 3 and 4), who need to start radiotherapy and/or chemotherapy, will be eligible for the protocol.

### 2.4. Intervention

Eligible patients will be included during a consultation with an investigator after verification of their compliance with the inclusion and non-inclusion criteria (and signing of informed consent).

A fasting blood sample will be taken on the start and end day of radiotherapy and during adjuvant chemotherapy (every 3 cycles). Follow-up monitoring will be performed for 9 months, with a blood sample taken every 3 months on the day of the follow-up MRI.

Pseudonymized blood samples will be stored at the Centre Jean PERRIN and then sent to the Hospices Civils de Lyon (France) for analysis. The blood samples will be analyzed to evaluate the plasma concentrations of hPG_80_ in pmol/L using the ELISA DxPG80 kit (BIODENA CARE, France).

The risks or constraints appear minimal for the subjects participating in the study since the only interventions are blood tests.

### 2.5. Measures of hPG80 levels in blood samples

The ELISA DxPG80 lab kit (BIODENA CARE, France) will be used to measure hPG_80_ levels in all plasma EDTA samples according to the manufacturer's instructions. The analytical performances of the kit have been described by Cappellini et al. (15). In brief, the limit of detection (LoD) is a hPG_80_ concentration of 1 pM and the limit of quantitation (LoQ) is a hPG_80_ concentration of 3.3 pM. The inter- and intra-assay coefficients of variation (CV%) are below 10%. No cross-reactivity has been detected with gastrin-17, Gastrin-Gly or CTFP (C-Terminus Flanking Peptide). No cross-reactivity has been detected with other blood biomarkers such as CA125, CEA or PSA. No interference has been detected with chemicals such as SN-38, 5-FU or triglycerides, cholesterol or hemoglobin.

### 2.6. Study procedures and participant timeline

The overview of study assessments and procedures are presented in Table 2.

**Table 2:** Data collection schedule.

		**Active treatment phase**			**Follow-up**		
	**Inclusion**	**Radiotherapy *(1st session)***	**Radiotherapy (Last session)**	**Adjuvant chemotherapy (Every 3 cycles)**	**3 months**	**6 months**	**9 months**
Information and consent	✓						
**Clinical evaluation**							
Medical & surgical history	✓						
History of the disease	✓						
Previous and concomitant treatments	✓	✓	✓	✓	✓	✓	✓
Toxicity evaluation (NCI CTCAE v5.0)	✓	✓	✓	✓	✓	✓	✓
**Biological examination**							
hPG80 plasma level assessment		✓	✓	✓	✓	✓	✓
**Tumor evaluation**							
MRI (rano evaluation)		✓	✓^*^	✓	✓	✓	✓

* Optional: If performed in standard practice.

### 2.7. Sample size

The PROGLIO study is a pilot, single-center, longitudinal study whose primary objective is to describe plasma hPG_80_ concentrations over time, in patients with high-grade brain tumors. At the present time, there are no published results on the expected distributions of plasma hPG_80_ concentrations during patient follow-up in the study population, hence the pilot aspect of our study, thus no power analysis is possible for the necessary sample size estimation. A sample size of 30 patients seems generally appropriate considering the state of recent literature. On the basis of preliminary data (currently confidential being consolidated for publication), we can at least estimate that the sample size of 30 patients should enable us to ensure 80% power (5% alpha risk, two-tailed test) to show an effect size of 0.75 of the difference between high-grade brain tumors and control values (healthy subjects) in the literature (16). The preliminary results were stratified by type of surgery (biopsy, partial resection, and total resection), and no gender differences were observed.

### 2.8. Data analysis

The main analysis consists of the descriptive analysis of plasma prograstin concentration values at each time point. For the third secondary objective, this analysis will be further developed by taking into account the longitudinal character of the data and by modeling data using mixed effects regression models, associated with graphical representations of individual trajectories.

The study of the relationship between hPG_80_ concentrations and tumor evaluation according to the RANO criteria (secondary objective 1) at different time points will be performed using generalized linear mixed models with response as the dependent variable, with hPG_80_ concentration as a fixed effect (as well as possibly other covariates), and with random effect calculations enabling the modeling of repeated data during follow-up.

The impact of radiotherapy on the variations in hPG_80_ concentrations (secondary objective 2) will be investigated using linear mixed models, followed, if relevant, by a *post-hoc* analysis by paired sample comparison tests.

### 2.9. Data management and monitoring

The data collected for the study will be recorded on an eCRF (Ennov Clinical software). The users with access to the data will be the investigators, the clinical research associates, the project leaders and the biostatisticians. They are authorized professionals and are subject to professional secrecy. The investigator will ensure the accuracy, completeness, and consistency of the data recorded (pseudonymized patient data) and of the provision of answers to data queries.

A clinical research associate mandated by the sponsor will perform regular monitoring reviews. The objectives will be to ensure the proper conduct of the study, the recording of the data generated in writing, and its documentation, storage and reporting, in accordance with the legislative and regulatory provisions in force. The follow-up reports will ensure traceability.

### 2.10. Consideration of risk of bias

The risk of selection bias should be limited by an exhaustive inclusion of eligible patients treated at the Centre Jean PERRIN during the study period. As recruitment is consecutive, a list of potentially eligible patients will be kept, in order to verify a posteriori the representativeness of the sample.

The existence of another undiagnosed cancer pathology also represents a risk of bias. In the event that this pathology is detected during the follow-up, a statistical analysis will be performed to evaluate the impact of the inclusion of these patients on the results obtained.

### 2.11. Trial status

The PROGLIO trial is currently recruiting. Participant recruitment began in February 2022 and recruitment is expected to finish in August 2023. The approved protocol is version 2, 20/10/2021.

## 3. Discussion

The investigation of blood biomarkers for high-grade glial tumors remains a major challenge in order to predict and monitor treatment response. Identify an accurate circulating biomarker is a highly rewarding area of interest in neuro-oncology. Differents methods have been examined (circulating tumor cells, cell-free DNA, microRNA, exosomes, and proteins) but no single biomarker test seemingly optimal at this time (19). This study, still at the proof-of-concept stage, could confirm that hPG_80_ is measurable in patients with high-grade glial tumors. If the main hypothesis of the PROGLIO trial is confirmed, further research will be required to demonstrate the clinical value of this blood biomarker. A study with a larger, multicentric enrolment would then enable its prognostic and diagnostic roles to be assessed, and to potentially highlight the usefulness of hPG_80_ as a blood biomarker.

## Ethics statement

The studies involving human participants were reviewed and approved by French Ethics Committee (Comité de protection des personnes Ouest III). The patients/participants provided their written informed consent to participate in this study.

## Author contributions

Conception and design: MC, XD, and IM. Principal investigators of the study: XD. Revision of the study design and protocol: MC, JP, and XD. Study coordination: MC and JP. Acquisition of data and patient recruitment and obtaining funding and supervision: MC and XD. Statistical analysis: IM. Drafting the manuscript: MC. Revision, adaptation and final approval of the manuscript: MC, JP, XD, and BV. All authors contributed to the article and approved the submitted version.
